# MSC-derived exosomes protect auditory hair cells from neomycin-induced damage via autophagy regulation

**DOI:** 10.1186/s40659-023-00475-w

**Published:** 2024-01-13

**Authors:** Huan Liu, Huijuan Kuang, Yiru Wang, Lili Bao, Wanxin Cao, Lu Yu, Meihao Qi, Renfeng Wang, Xiaoshan Yang, Qingyuan Ye, Feng Ding, Lili Ren, Siying Liu, Furong Ma, Shiyu Liu

**Affiliations:** 1 Department of Otolaryngology Head and Neck Surgery, Peking University Third Hospital, Beijing, China; 2 State Key Laboratory of Oral & Maxillofacial Reconstruction and Regeneration, National Clinical Research Center for Oral Diseases, Shaanxi International Joint Research Center for Oral Diseases, Center for Tissue Engineering, School of Stomatology,, The Fourth Military Medical University, Xi’an, Shaanxi China; 3 Anesthesiology Department, Seventh Medical Center of PLA General Hospital, Beijing, China; 4 Department of Periodontology, School and Hospital of Stomatology, Cheeloo College of Medicine, Shandong University and Shandong Key Laboratory of Oral Tissue Regeneration and Shandong Engineering Laboratory for Dental Materials and Oral Tissue Regeneration and Shandong Provincial Clinical Research Center for Oral Diseases, Jinan, Shandong China; 5 Department of Otolaryngology-Head and Neck Surgery, Xijing Hospital, Air Force Military, Xi’an, Shaanxi China; 6 School of Stomatology, Stomatological Hospital, Southern Medical University, Guangzhou, China; 7 State Key Laboratory of Oral & Maxillofacial Reconstruction and Regeneration, National Clinical Research Center for Oral Diseases, Shaanxi Key Laboratory of Stomatology, Digital Dentistry Center, School of Stomatology, The Fourth Military Medical University, Xi’an, Shaanxi China

**Keywords:** Mesenchymal stem cell, Exosome, Hair cell, Neomycin, Aminoglycoside, Autophagy, Endocytosis

## Abstract

**Background:**

Sensorineural hearing loss (SNHL) poses a major threat to both physical and mental health; however, there is still a lack of effective drugs to treat the disease. Recently, novel biological therapies, such as mesenchymal stem cells (MSCs) and their products, namely, exosomes, are showing promising therapeutic potential due to their low immunogenicity, few ethical concerns, and easy accessibility. Nevertheless, the precise mechanisms underlying the therapeutic effects of MSC-derived exosomes remain unclear.

**Results:**

Exosomes derived from MSCs reduced hearing and hair cell loss caused by neomycin-induced damage in models in vivo and in vitro. In addition, MSC-derived exosomes modulated autophagy in hair cells to exert a protective effect. Mechanistically, exogenously administered exosomes were internalized by hair cells and subsequently upregulated endocytic gene expression and endosome formation, ultimately leading to autophagy activation. This increased autophagic activity promoted cell survival, decreased the mitochondrial oxidative stress level and the apoptosis rate in hair cells, and ameliorated neomycin-induced ototoxicity.

**Conclusions:**

In summary, our findings reveal the otoprotective capacity of exogenous exosome-mediated autophagy activation in hair cells in an endocytosis-dependent manner, suggesting possibilities for deafness treatment.

**Supplementary Information:**

The online version contains supplementary material available at 10.1186/s40659-023-00475-w.

## Background

Disabling hearing loss, which affects approximately 5% of the global population (approximately 430 million people), is one of the most common sensory disorders in humans and has attracted increasing attention worldwide [1]. Genetic mutations, noise exposure, ototoxic drugs, and aging can contribute to sensorineural hearing loss (SNHL) [2]. Treatment of SNHL remains a challenge since hair cells, which are terminally differentiated cells, are difficult to regenerate and recover once they are damaged [3]. Current treatments rely heavily on devices such as hearing aids and cochlear implants, and there is no FDA-approved drug for the condition [4].

Because there are few treatments, the study of biological alternatives, such as gene therapy, growth factor therapy, molecular strategies, and stem-cell therapy have been explored [2, 3, 5]. In the case of stem cell therapy, mesenchymal stem cells (MSCs) are suitable candidates for cell-based medical therapies and they have exhibited remarkable potential to repair or regenerate damaged cochlear hair cells and rescue hearing loss in SNHL animal models [6, 7]. However, the underlying therapeutic mechanisms are not fully understood. Accumulating evidence indicates that MSCs exert ultimate therapeutic effects by producing exosomes [8]. Exosomes, extracellular vesicles with a diameter of 30–150 nm, can act as messengers carrying various bioactive payloads, including proteins, lipids, and nucleic acids, to regulate pathways associated with inflammation, proliferation, apoptosis, differentiation, and metabolism [9–11]. To date, exosomes released by inner ear tissue have been shown to mediate nonautonomous cell survival signaling and protect sensory hair cells against lethality-inducing stress by carrying heat shock protein 70 (HSP70) [12, 13]. This finding suggests that exosomes derived from MSCs may be valuable therapeutic agents that prevent or reverse hearing loss. However, the underlying therapeutic mechanisms of exosome actions are not completely understood.

Autophagy is an orderly cellular process through which unnecessary or abnormal cellular components are degraded through a highly conserved catabolic pathway in all eukaryotic cells [14]. This pathway acts as a protective mechanism for cochlear hair cells against damage induced by noise exposure, ototoxic drugs, and aging [15, 16]. Studies have demonstrated that autophagy and exosomes may be regulated concomitantly or reciprocally depending to the cellular conditions, and exosomes have been shown to regulate autophagy, an intracellular process [17]. In addition, to generate biological effects, exosomes need to fuse with the cell membrane of recipient cells or be internalized by recipient cells through endocytosis, and this biological process may affect the biological functions of the recipient cell. Interestingly, accumulating evidence indicates that endocytosis is critical and necessary for autophagy [17, 18]. Therefore, it is reasonable to investigate whether exosomes could induce endocytosis to mediate biological effects on hair cells’ autophagy.

In this study, we selected a neomycin-induced SNHL model to test this hypothesis, since the impairment of hair cells induced by ototoxic drugs is one of the major causes of SNHL, and aminoglycoside antibiotics, such as neomycin, are among the major classes of ototoxic drugs. Among different tissue sources of MSCs, umbilical cord (UC) tissue MSCs has the characteristics of noninvasive harvesting procedure, easily expanding in vitro, less cellular aging and ethical issues. Moreover, umbilical cord-mesenchymal stem cells (UC-MSCs) have demonstrated promising potential for the treatment of different disease due to their differentiation capacity, immune regulation, paracrine and anti-inflammatory effects [19, 20]. Preclinical studies have demonstrated that umbilical cord-mesenchymal stem cells (UC-MSCs) can significantly decrease the hearing threshold and increase the number of outer hair cells in an SNHL model [21, 22]. Therefore, UC-MSCs, one of the most widely applied MSCs in clinical trials, were used to produce exosomes, the function of which was explored in our study [23]. In the present study, we investigated the protective effect of MSC-derived exosomes on hair cells in a neomycin-induced SNHL model and the potential role of these vesicles in enhancing autophagy activity in hair cells. We found that exosomes derived from MSCs protected hair cells from neomycin-induced damage and promoted autophagy. Mechanistically, exosomes promoted the expression of endocytic genes and the formation of endosomes, which are required for exosome-induced autophagy activation, thereby rescuing the loss of cochlear hair cells and ameliorating hearing damage.

## Results

### Exosomes derived from UC-MSCs reduced hearing and hair cell loss damaged by neomycin in vivo

**Fig. 1:**
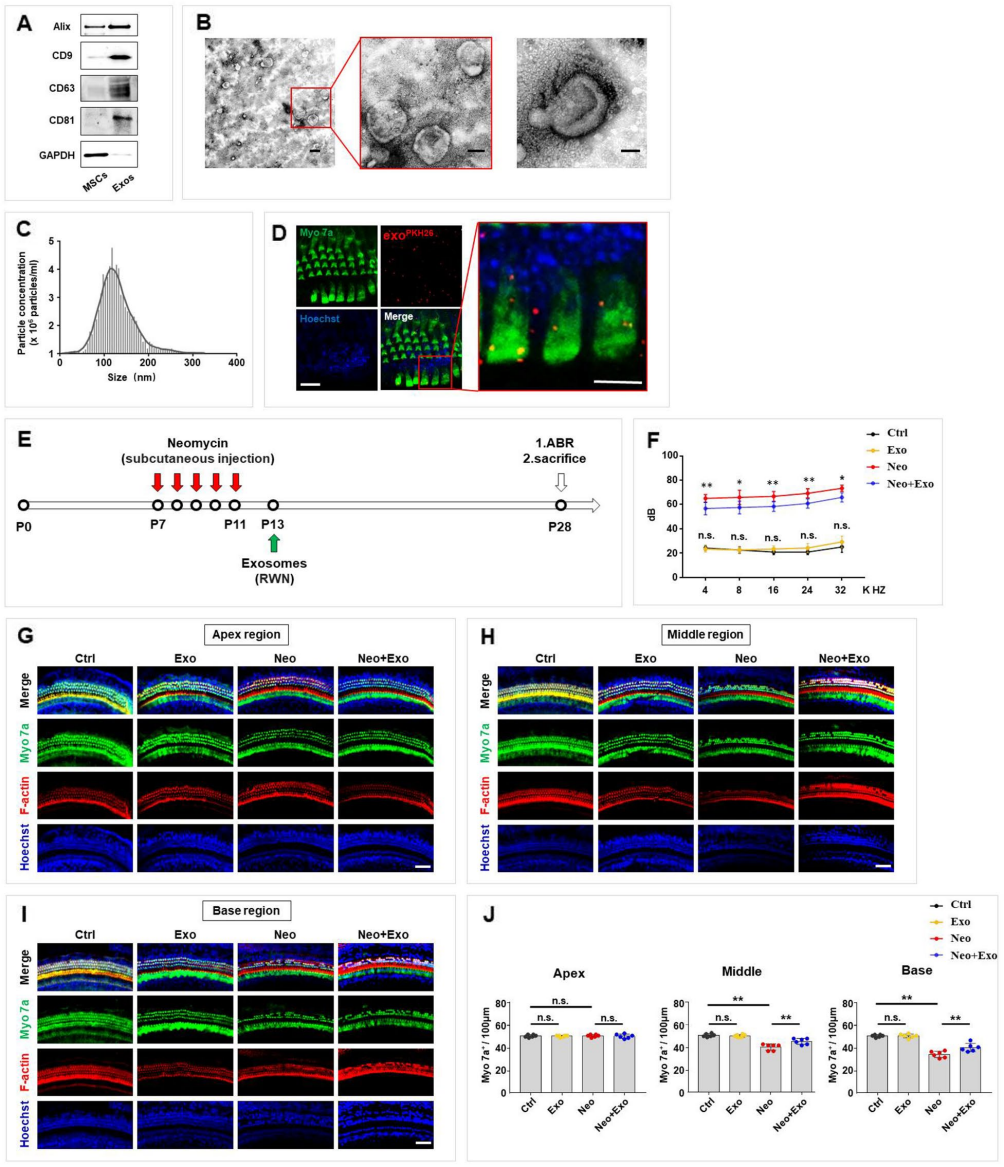
Characterization of exosomes and their capacity to reduce hearing loss and decrease hair cell loss after neomycin damage in mice. **A** Western blot analysis of exosome markers Alix, CD9, CD63, and CD81 in MSCs and MSC-derived exosomes, and the negative intracellular protein marker GAPDH. **B** Transmission electron microscope (TEM) analysis of MSC-derived exosomes. Scale bar, 200 nm (left), 100 nm (middle) and 50 nm (right). **C** Size distribution of exosomes measured by nanoparticle tracking analysis (NTA). **D** Confocal microscopy image showing exosomes internalization by hair cells in vitro. Scale bar, 20 μm (left) and 10 μm (right). (**E**) Schematic diagram of animal experiment workflow, round window niche (RWN). **F** Analysis of ABR thresholds in mice treated with neomycin (Neo, 200 mg/kg for five consecutive days) and/or exosomes (Exo, 20 μg in 10 μl PBS). n = 6 mice **G**–**I** Immunofluorescence staining with Myo 7a (green), F-actin (red) and Hoechst (blue) in the apical (**G**), middle (**H**), and basal (**I**) turns of the cochleae from different groups. Scale bars, 50 μm. **J** Quantification of Myo 7a-positive hair cells in the apical, middle, and basal turns of the cochleae. The results were representative of the data generated in at least three independent experiments. The data were presented as mean ± s.d. n.s., not significant; *P < 0.05; **P < 0.01 by one-way ANOVA (**F**, **J**)

First, we hypothesized that exosomes derived from UC-MSCs exert a protective effect against neomycin-induced ototoxicity in mice. The cell supernatants of MSCs were collected, and exosomes were isolated through a sequential centrifugation procedure. The purified UC-MSC-derived exosomes were characterized by immunoblotting, transmission electron microscopy (TEM), and nanoparticle tracking analysis (NTA). A western blot analysis showed that the exosome surface markers Alix, CD9, CD63, and CD81 were more abundant in the exosomal lysate compared to UC-MSC lysate (Fig. 1A). The exosomal lysate was probed for GAPDH content, which was the negative control for intracellular protein, and no positive staining was observed (Fig. 1A). TEM and NTA were used to evaluate the morphology and size distribution of the exosomes. As shown in Fig. 1B and C, the exosomes typically displayed a round shape and a cup-shaped appearance (Fig. 1B), and they composed a homogenous population with sizes ranging primarily between 30 and 150 nm in diameter (Fig. 1C). Then, with cultured cochlear explants, we confirmed that exosomes were internalized by hair cells in co-culture, as shown by confocal microscopy (Fig. 1D).

To assess their therapeutic benefits further, exosomes were injected into neomycin-induced SNHL mice through the round window niche (RWN). Hearing function was evaluated using auditory brainstem response (ABR) measurements after different treatments. The workflow of the animal experiments is shown in Fig. 1E. The hearing threshold in the exosome-alone group was similar with that in the control group (Fig. 1F). The hearing threshold significantly increased in the neomycin-injected group compared with that in control, while injection of exosomes into the RWN of neomycin-exposed mice significantly reduced the hearing threshold at 4, 8, 16, 24, and 32 kHz (Fig. 1F). In addition, an immunofluorescence assay was performed to measure the loss of hair cells in cochlear tissue labeled by Myo 7a (myosin 7a) and F-actin. The results revealed that exosome-alone-treated mice showed no hair cells loss in all turns while neomycin-treated mice showed a significant loss of Myo 7a-positive hair cells in the middle and basal regions. In contrast, the administration of exosomes in neomycin-injected mice significantly reduced the loss of Myo 7a-positive hair cells in the middle and basal regions of the cochlear tissues (Fig. 1G, H, I, J). These results suggested that exosomes attenuated hearing loss and associated hair cell loss in neomycin-induced SNHL mice.

### Exosomes derived from UC-MSCs protected hair cells against neomycin-induced damage in vitro

**Fig. 2:**
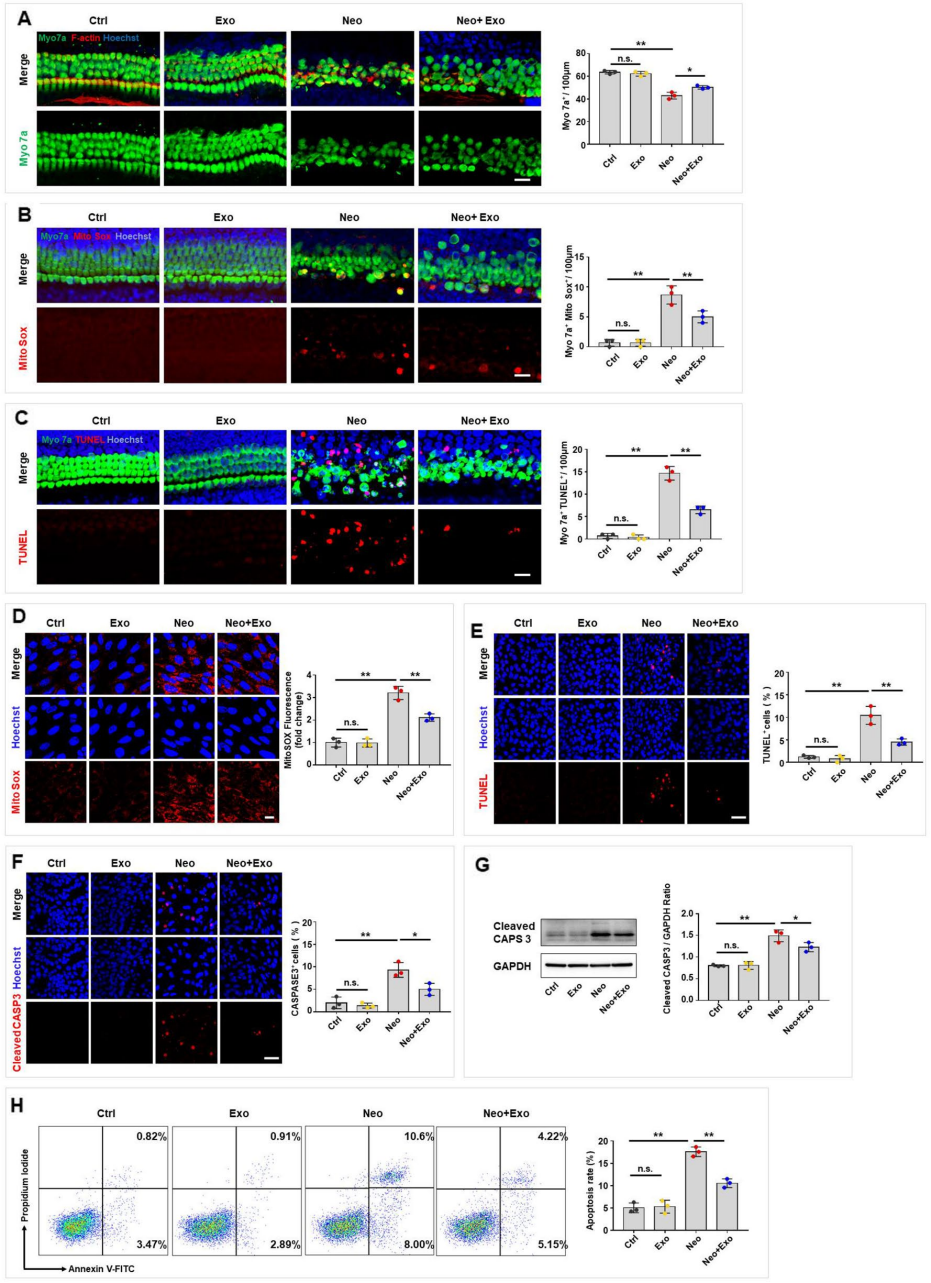
Exosomes protected hair cells against neomycin-induced damage in vitro. **A**–**C** Immunofluorescence staining was performed after neomycin damage (0.5 mM, 24 h) then with/without exosomes treatments (30 μg/ml, 24 h) in the middle turn of cochleae. **A** Immunofluorescence staining with Myo 7a (green), F-actin (red) and Hoechst (blue) after different treatments. Quantification of Myo 7a-positive hair cells per 100 μm in the middle turn of different groups. Scale bar, 20 µm. **B** Immunofluorescence staining with Myo 7a (green), Mito-SOX (red) and Hoechst (blue) after different treatments. The numbers and proportions of Mito-SOX and Myo 7a double-positive cells were quantified. Scale bar, 20 µm. **C** Immunofluorescence staining with Myo 7a (green), TUNEL (red), and Hoechst (blue) after different treatments. The numbers of TUNEL and Myo 7a double-positive cells were quantified. Scale bar, 20 µm. **D**–**H** HEI-OC1 cells were treated with neomycin (2 mM, 24 h), then with/ without exosomes (30 μg/ml, 24 h) treatment. **D** HEI-OC1 cells were labeled with Mito-SOX (red) and Hoechst (blue) and the relative fluorescence intensity was quantified. Scale bar, 20 µm. **E** TUNEL and Hoechst double staining and **F** Cleaved CASP3 and Hoechst double staining were performed to detect the percentage of apoptotic HEI-OC1 cells. Scale bar, 50 µm. **G** Cleaved CASP3 expression was detected by western blot in HEI-OC1 cells and was quantified by ImageJ software. **H** Analysis of apoptotic HEI-OC1 cells by flow cytometry. The results were representative of the data generated in at least three independent experiments. The data were presented as mean ± s.d. n.s., not significant; *P < 0.05; **P < 0.01 by one-way ANOVA (**A**-**H**)

As described, exosomes released by MSCs reduced the hearing threshold and hair cell loss in neomycin-induced SNHL mice. We further examined the impact of exosomes on cell survival, the oxidative stress level and the apoptosis rate of hair cells in cultured cochlear explants and HEI-OC1 cells in vitro. In the cultured cochlear explants, cell survival was assessed by counting the number of Myo 7a-positive hair cells in 100 μm lengths on all turns of the cochleae after neomycin damage. The results showed that hair cell loss in the middle and basal turns of the cochlea was increased after treatment with 0.5 mM neomycin for 24 h, but this loss was significantly attenuated in a dose-dependent manner after treatment with exosomes (Additional file 1: Fig. S1). As a concentration of 30 μg/ml exosomes, the cochlear sample from the middle turn was the most significantly protected, 30 μg/ml exosomes were applied in the subsequent experiments, and only the middle turn was investigated. As indicated by immunofluorescence assay and cell counting, exosomes did not affect hair cell survival. However, hair cell survival was reduced after neomycin exposure. Interestingly, exosomes were found to increase hair cell survival following neomycin-induced damage (Fig. 2A). Additionally, we used Mito-SOX Red, a redox fluorophore that enables selective detection of mitochondrial superoxide, to evaluate mitochondrial reactive oxygen species (ROS) levels in the cochleae. The results demonstrated that exosomes did not alter the level of ROS. However, exosomes reduced the oxidative stress caused by neomycin damage in cochlear hair cells (Fig. 2B). Furthermore, terminal deoxynucleotidyl transferase dUTP nick end labeling (TUNEL) assays were performed to evaluate hair cell apoptosis. The results showed that exosomes did not induce an increase in the apoptosis rate of hair cells, while neomycin significantly increased the apoptosis rate of hair cells. However, exosomes ameliorated apoptosis in cochlear hair cells with neomycin damage (Fig. 2C).

In addition, we investigated the effect of exosomes on the oxidative stress level and apoptosis rate in HEI-OC1 cell under normal conditions and after neomycin-induced damage. Mito-SOX Red was used to assess mitochondrial ROS levels in the HEI-OC1 cells. An immunofluorescence assay showed no significantly increase after exosome-alone treatment while a significant increase in ROS levels after neomycin treatment. However, exosomes reduced neomycin-induced ROS accumulation in HEI-OC1 cells (Fig. 2D). To assess the apoptosis rate, we performed TUNEL staining to identify apoptotic HEI-OC1 cells after neomycin and/or exosome treatments. The results showed that the proportion of TUNEL-positive HEI-OC1 cells in the exosome-alone-treated group was similar to the control group, while the proportion of TUNEL-positive HEI-OC1 cells in the neomycin-treated group was higher than that in the control group. Interestingly, exosome treatment significantly reduced the proportion of TUNEL-positive cells in the neomycin-treatment group (Fig. 2E). Next, immunofluorescence and western blot assays were performed to measure the proportion of cleaved CASP3-positive cells among HEI-OC1 cells and the level of cleaved CASP3 protein expression in these cells, respectively. The results showed that exosome-alone did not alter the proportion of cleaved CASP3-positive cells in the cell population and the protein expression of cleaved CASP3, while the exosome treatment significantly reduced the proportion of cleaved CASP3-positive cells in the cell population and the protein expression of cleaved CASP3 after neomycin-induced damage in HEI-OC1 cells (Fig. 2F, G). Additionally, we used Annexin V and propidium iodide (PI) assay, in which propidium iodide is used to label dead cells and ANXA5/Annexin V is used to label cells undergoing apoptosis, and analyzed the number of cells undergoing apoptosis by flow cytometry. The results demonstrated that exosome-alone treatment did not increase the proportion of dead and apoptotic cells, while neomycin treatment significantly increased the proportion of dead and apoptotic cells compared to the number in the control group. However, the proportion of neomycin-exposed apoptotic cells after exosome treatment was significantly reduced compared to that in the neomycin damage group (Fig. 2H). These results suggested that exosomes protected hair cells against neomycin-induced damage by promoting cell survival, reducing mitochondrial ROS levels, and decreasing the apoptosis rate in both cultured cochlear explants and HEI-OC1 cells in vitro.

### Exosomes increased autophagy in hair cells

**Fig. 3:**
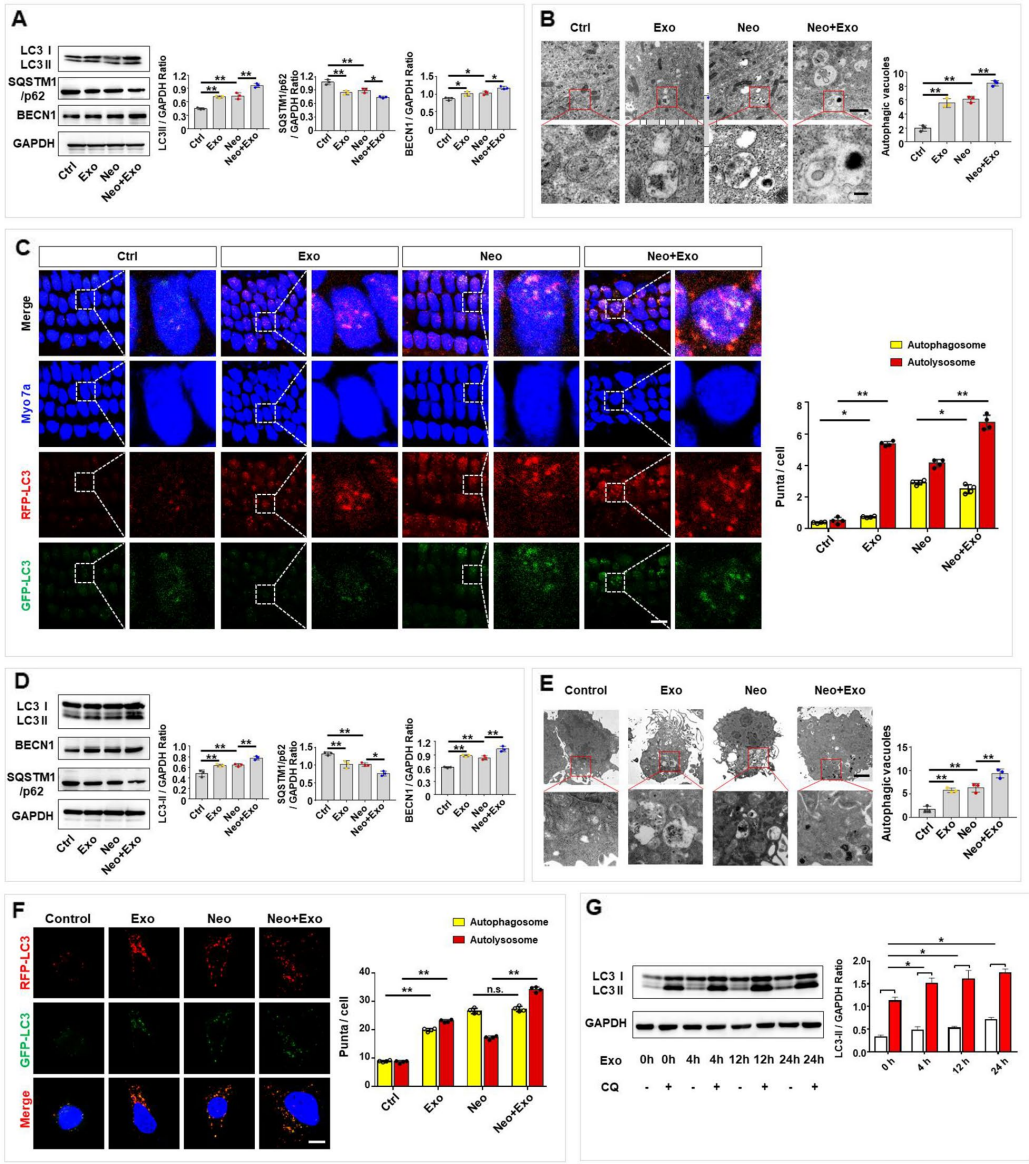
Exosomes improved autophagy in cochlear hair cells and HEI-CO1 cells. **A** Expression of LC3, SQSTM1/p62 and BECN1 in cochlear explants treated with neomycin (0.5 mM, 24 h) and/or exosomes (30 μg/ml, 24 h) was evaluated by western blot and quantified by ImageJ software. **B** TEM analysis was used to evaluate autophagy in cochlear hair cells, and the numbers of autophagic vacuoles were quantified. Scale bar, 1 µm (up), 200 nm (down). **C** Immunofluorescence staining with Myo 7a (blue) in cochleae from CAG-RFP-EGFP-LC3 mice. The numbers of autophagosomes (yellow) and autolysosomes (red-only puncta) per cell were quantified. Scale bar, 10 µm. **D** Expression of LC3, SQSTM1/p62 and BECN1 in HEI-OC1 cells treated with neomycin and/or exosomes was evaluated by western blot and quantified by ImageJ software. **E** Autophagy in HEI-OC1 cells was detected by TEM, and the numbers of autophagic vacuoles were quantified. Scale bar, 2 µm (up), 500 nm (down). **F** HEI-OC1 cells were infected with mRFP-GFP-LC3 (tfLC3) and then treated with neomycin and/or exosomes. The numbers of autophagosomes (yellow) and autolysosomes (red-only puncta) per cell were quantified. Scale bar, 10 µm. **G** Autophagy flux was evaluated by western blotting for LC3 with or without CQ (20 μM). Autophagy flux assay was used in HEI-OC1 cells treated with exosomes at different time points, and LC3-II (CQ-Ctrl) was quantified by ImageJ software. The results were representative of the data generated in at least three independent experiments and presented as mean ± s.d. n.s., not significant; *P < 0.05; **P < 0.01 by one-way ANOVA (**A**-**G**)

We then explored whether autophagy activation in hair cells was increased by exosome treatment. Autophagy activation and autophagic flux in cultured cochlear explants and HEI-OC1 cells were assessed by western blotting, TEM and a tandem fluorescence mRFP-GFP-LC3 reporter system assay. The expression of autophagy-associated protein LC3, SQSTM1/p62 and BECN1 in cochleae was measured by western blotting. The results showed that the protein expression level of the autophagy-associated protein LC3-II and BECN1 was upregulated after treatment with exosomes or neomycin compared with that in the control samples, and the expression levels were further increased by exosome treatment in neomycin-exposed explants (Fig. 3A). The protein expression level of SQSTM1/p62 decreased compared with that in the control, and the expression levels were further reduced by exosome treatment in neomycin-exposed explants (Fig. 3A). In addition, TEM was used to observe the autophagic vacuoles [24] (including the autophagosome, amphisome, and autolysosome) in the cultured cochlear explants. The results revealed significantly more autophagic vacuoles after exosome or neomycin treatment compared with the number in the control, and the number of autophagic vacuoles was further increased by exosome treatment following neomycin exposure (Fig. 3B). CAG-RFP-EGFP-LC3 mice were used to confirm changes in autophagic flux after exosome treatment and/or neomycin exposure. Cochleae were dissected from P2 CAG-RFP-EGFP-LC3 mice and immunolabeled with the hair cell marker Myo 7a. Quantification of LC3 puncta in each hair cell showed that the number of red-only puncta (marking autolysosomes) was significantly increased compared with that of yellow puncta (marking autophagosomes) in hair cells after exosome treatment compared with the numbers in the control, and these punctate numbers were further increased by exosome treatment after neomycin exposure, as the numbers of red-only (marking autolysosomes) and yellow puncta (marking autophagosomes) were increased (Fig. 3C). The results showed that exosome treatment promoted autophagosome–lysosome fusion under normal conditions and after neomycin-induced autophagy activation.

LC3, SQSTM1/p62 and BECN1 expression in HEI-OC1 cells was then assessed by western blotting. Consistent with previous data from cultured cochlear explants, LC3-II and BECN1 expression was upregulated after treatment with exosomes or neomycin compared to that in the control, and the expression level was further increased by exosome treatment after neomycin exposure (Fig. 3D). The protein expression level of SQSTM1/p62 decreased after treatment with exosomes or neomycin compared with that in the control, and the expression levels were further reduced by exosome treatment in neomycin-exposed cells (Fig. 3D). Next, TEM images showed significantly more autophagic vacuoles in the exosome or neomycin treatment group than in the control group, and the number of autophagic vacuoles was further increased by exosome treatment even after neomycin-induced increase (Fig. 3E). To determine whether exosomes can increase autophagic flux in HEI-OC1 cells, a tandem fluorescence mRFP-GFP-LC3 reporter system was used to monitor autophagic flux. The results showed that exosomes significantly increased the number of red-only puncta, while more yellow puncta than red-only puncta were counted after neomycin treatment. Additionally, exosome treatment increased the number of red-only puncta after HEI-OC1 cells were exposed to neomycin, suggesting the formation of autolysosomes (Fig. 3F). Furthermore, autophagic flux was analyzed by western blot analysis. Chloroquine (CQ) was used to inhibit autophagy by blocking autophagosome–lysosome fusion, thereby inducing the accumulation of autophagosomes. LC3 expression was measured by western blot in exosome-treated HEI-OC1 cells with or without CQ. Consistent with the fluorescence data, exosome treatment increased the LC3-II level at 4 h, 12 h and 24 h in HEI-OC1 cells treated with CQ (Fig. 3G). In summary, these results illustrate the ability of exosomes to increase autophagic flux in cultured cochlear explants and HEI-OC cells.

### Autophagy was necessary for exosome-induced hearing protection in vivo

**Fig. 4:**
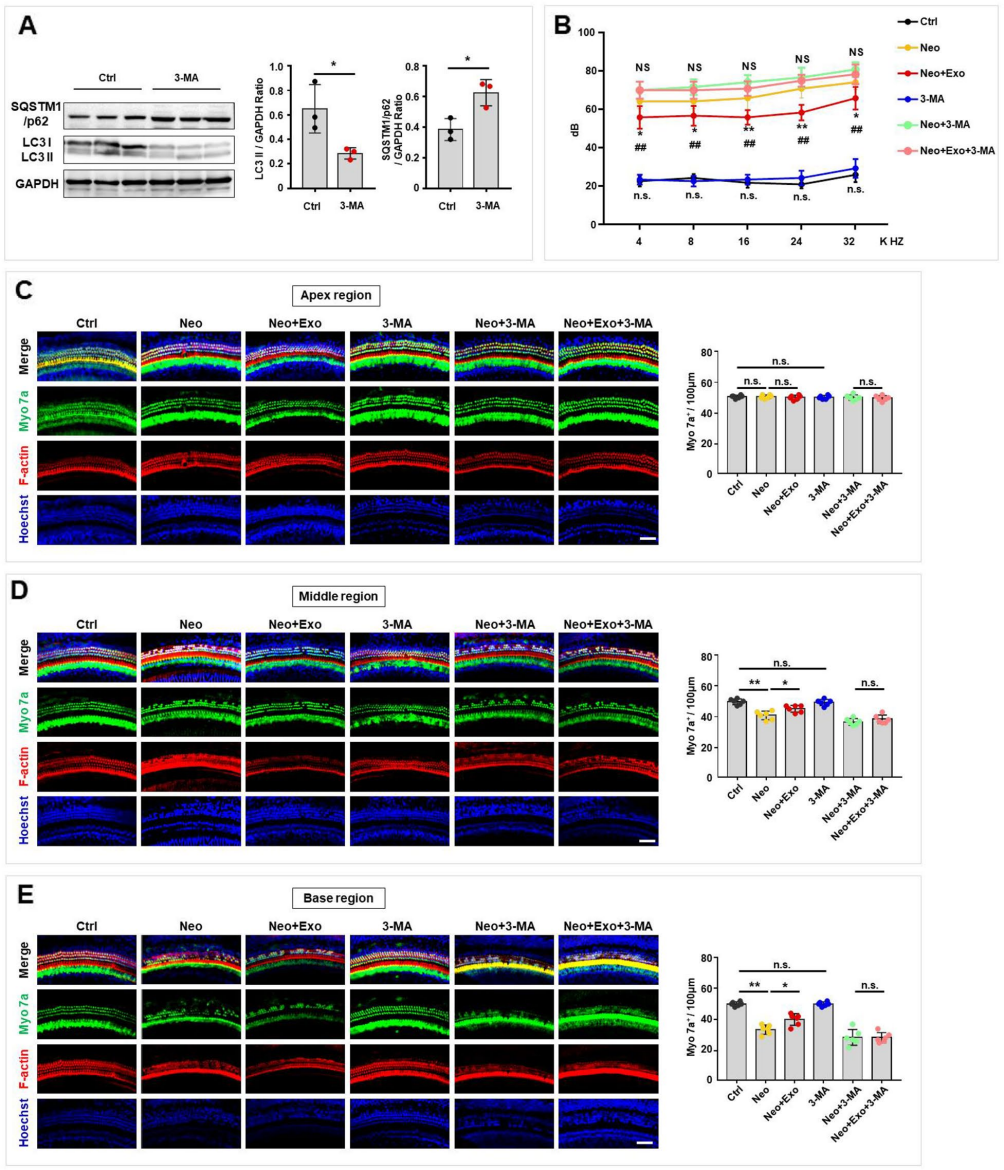
Autophagy was required for exosome-mediated hearing functional recovery in the neomycin-induced SNHL mice model. **A** The expression levels of LC3 and SQSTM1/p62 in cochlear tissues were evaluated by western blot after 3-MA treatment and LC3-II and SQSTM1/p62 was quantified by ImageJ software. **B** ABR thresholds were analyzed in mice with different treatment. Untreated control (Ctrl, black), neomycin alone (Neo, yellow), neomycin and exosome treatment (Neo + Exo, red) 3-MA alone (3-MA, blue), neomycin and 3-MA (Neo + 3-MA, green), and neomycin, exosome and 3-MA (Neo + Exo + 3-MA, pink). n = 6 mice. *##p* < 0.01 Ctrl vs. Neo; *p < 0.01; **p < 0.01 Neo vs. Neo + Exo; n.s., not significant Ctrl vs. 3-MA; NS, not significant Neo + 3-MA vs. Neo + Exo + 3-MA by one-way ANOVA. **C**–**E** Immunofluorescence staining with Myo 7a (green), F-actin (red), and Hoechst (blue) in the apical (**C**), middle (**D**), and basal (**E**) turns of cochleae from different groups. Scale bars = 50 μm. Quantification of Myo 7a-positive hair cells in the apical (**C**), middle (**D**), and basal (**E**) turns of the cochleae from different groups. The results were representative of the data generated in at least three independent experiments and data were presented as mean ± s.d. n.s., not significant; *P < 0.05; **P < 0.01 by Student’s t-test (**A**) or by one-way ANOVA (**B**–**E**)

Exosomes promoted autophagy activation in hair cells in both cultured cochlear explants and HEI-OC1 cells. To further explore whether autophagy regulation is necessary for exosome-induced inner ear protection in vivo, 3-methyladenine (3-MA), an autophagy antagonist that inhibits Class III phosphatidylinositol 3-kinase (PI3K), was first used. A western blot analysis showed that 3-MA administration effectively decreased LC3-II levels and increased SQSTM1/p62 levels, thereby inhibiting autophagy in mouse cochlear tissue (Fig. 4A). Next, hearing ability was assessed after exosome treatment with or without autophagy inhibition. Consistent with the aforementioned results, the neomycin-exposed group exhibited increased hearing threshold (Fig. 4B) and decreased hair cell survival (Fig. 4C-E) compared with that in the control group. Besides, the exosome-treated group showed higher hearing function after neomycin injection than the neomycin-exposed group, as assessed by a decreased hearing threshold (Fig. 4B) and increased hair cell survival (Fig. 4C-E). Moreover, the autophagy inhibition group showed an attenuated therapeutic effect of exosomes on the recovery of hearing threshold (Fig. 4B). Additionally, the beneficial effects of exosomes on hair cell survival (Fig. 4C–E) were attenuated when autophagy was inhibited by 3-MA. However, the autophagy inhibition by 3-MA did not affect the heating threshold (Fig. 4B) and hair cells survival (Fig. 4C–E) compare with that in the control group. These results showed that autophagy inhibition with 3-MA blocked the therapeutic effect generated by exosomes and that autophagy was necessary for exosome-induced hearing protection.

### Autophagy was required for exosome-mediated protection of hair cells

**Fig. 5:**
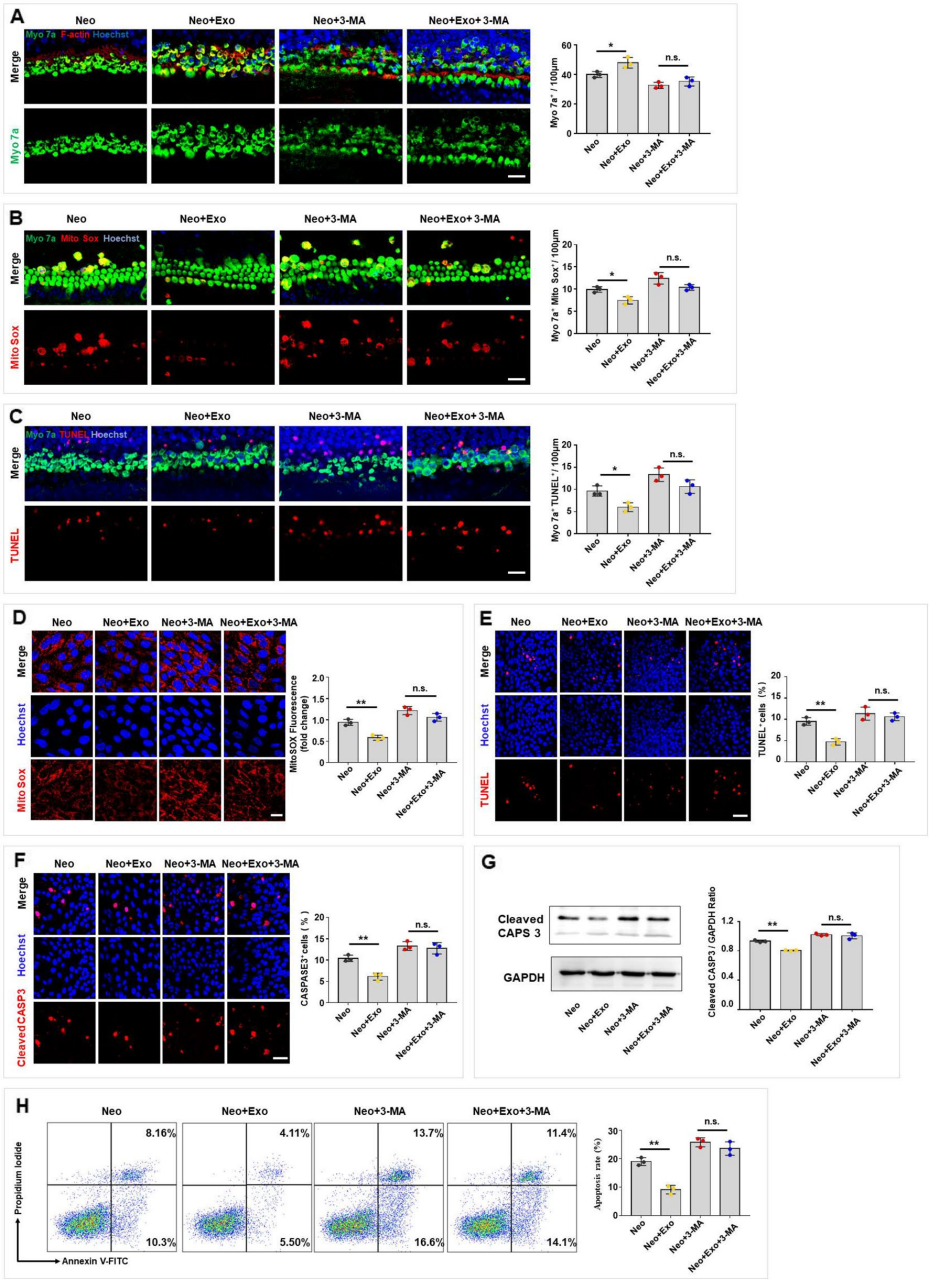
Autophagy was necessary for exosome-mediated otoprotection. **A**–**C** Cochlear explants were treated with 3-MA (5 mM) for 16 h. Cell survival, oxidative stress and apoptosis of hair cells were respectively detected by phalloidin staining (**A)**, Mito-SOX Red (**B)**, and TUNEL assay (**C)** following co-culture with exosomes after neomycin exposure. The number of F-actin and Myo 7a double-positive cells, Mito-SOX and Myo 7a double-positive cells and TUNEL and Myo 7a double-positive cells per 100 μm in the middle turn of different groups were quantified. Scale bar, 20 µm. **D**–**H** HEI-OC1 cells were treated with 3-MA (5 mM) for 16 h to inhibit autophagy. **D** HEI-OC1 cells were labeled with Mito-SOX (red), and the relative fluorescence intensity was quantified after different treatments. Scale bar, 20 µm. **E** TUNEL and Hoechst double staining and **F** Cleaved CASP3 and Hoechst double staining were performed to detect the percentage of apoptotic HEI-OC1 cells after different treatments. Scale bar, 50 µm. **G** Cleaved CASP3 expression was detected by western blot in HEI-OC1 cells treated with exosomes and/or 3-MA following neomycin insults and was quantified by ImageJ software. **H** Analysis of apoptotic HEI-OC1 cells by flow cytometry after different treatments. The results were representative of the data generated in at least three independent experiments. The data were presented as mean ± s.d. n.s., not significant; *P < 0.05; **P < 0.01 by one-way ANOVA (**A**–**H**)

To determine whether autophagy is necessary for exosome-mediated protection of hair cells, 3-MA was used to inhibit autophagy in vitro. Cell survival, the oxidative stress level and the apoptosis rate of hair cells were assessed in cultured cochlear explants and HEI-OC1 cells. Consistent with the aforementioned results, exosome treatment increased cell survival in neomycin-treated cochleae, as assessed by immunofluorescence and shown by the number of Myo 7a-positive cells per 100 μm (Fig. 5A), while autophagy inhibition attenuated the exosome-mediated therapeutic effects on promoting hair cell survival (Fig. 5A). Additionally, the protective effects of exosomes conferred by mitochondrial ROS level reduction (Fig. 5B) and apoptosis rate reduction (Fig. 5C) were attenuated when autophagy was inhibited by 3-MA.

Similar results were observed in HEI-OC1 cells. In the presence of 3-MA, exosomes failed to protect HEI-OC1 cells from neomycin-induced damage, including oxidative stress, as assessed by Mito-SOX Red staining (Fig. 5D), and apoptosis, as assessed by TUNEL assays (Fig. 5E), cleaved CASP3 staining (Fig. 5F), western blotting (Fig. 5G), and flow cytometry (Fig. 5H). Moreover, autophagy inhibition was also performed by knocking down the autophagy-related gene *Atg5* in HEI-OC1. As the results shown, the protein expression level of ATG5, LC3 was significantly reduced with SQSTM1/p62 increasing after knockdown of *Atg5*, which was assessed by western blot (Additional file 1: Fig. S2A). Then, exosomes treatment decreased ROS level in neomycin-treated HECI-OC1, as assessed by Mito-SOX Red staining (Additional file 1: Fig. S2B), while inhibition of autophagy by knocking down *Atg5* weakened the exosome-mediated reduction of ROS levels (Additional file 1: Fig. S2B). Additionally, the protective effects of exosomes were confirmed by reduced apoptosis rate assessed by TUNEL assays (Additional file 1: Fig. S2C), cleaved CASP3 staining (Additional file 1: Fig. S2D), western blotting (Additional file 1: Fig. S2E), and flow cytometry (Additional file 1: Fig. S2F) in HEI-OC1, while the efficacy were attenuated when autophagy was inhibited by knocking down *Atg5* (Additional file 1: Fig. S2C, D, E, F). Taken together, these data indicated that autophagy is essential for exosome-mediated protection in both cultured cochlear explants and HEI-OC1 cells.

### Exosomes upregulated autophagy by promoting endocytosis in hair cells

**Fig. 6:**
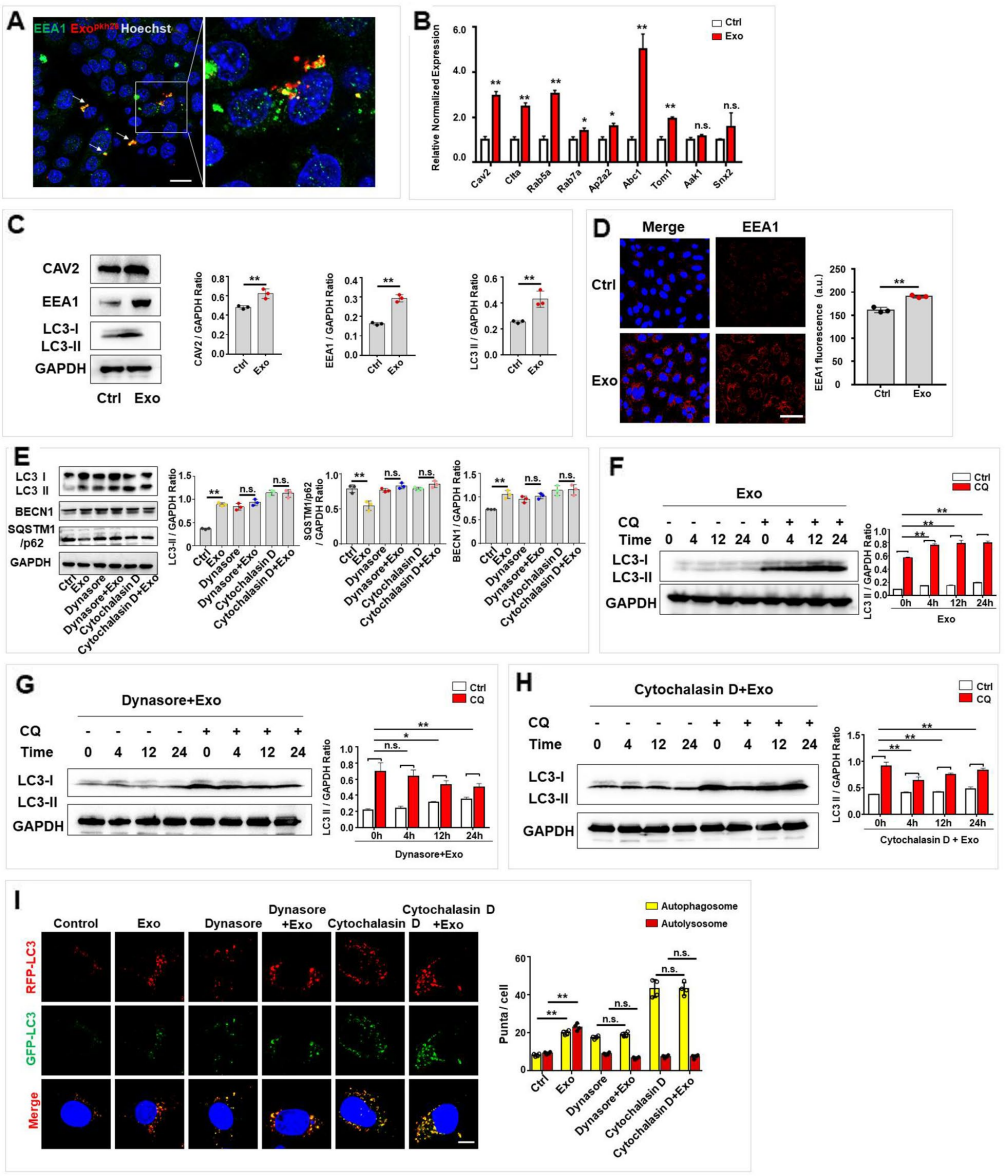
Exosomes promoted endocytosis, which is required for exosomes to upregulate autophagy of hair cells. **A** PKH26-labeled exosomes co-localized with endosomes, which were immunofluorescence stained with EEA1 in HEI-OC1. Scale bar: 20 µm. **B** RT-qPCR was performed to detect the endocytosis-associated mRNA levels of HEI-OC1 cells after treatment with exosomes (30 μg/ml, 24 h). **C** The protein expression of CAV2, EEA1 and LC3 was detected by western blot and was quantified by ImageJ software. **D** The expression of EEA1 was detected by immunofluorescence staining, and the fluorescence intensity was quantified by ImageJ software. Scale bar, 50 μm. **E** Expression of LC3, SQSTM1/p62 and BECN1 were detected by western blot after exosome treatment with or without pre-treatment of dynasore (80 μM, 4 h) or cytochalasin D (2 μM, 30 min) and were quantified by ImageJ software. **F** Autophagy flux assay of HEI-OC1 cells treated with exosomes without endocytosis inhibition, and LC3-II (CQ-Ctrl) was quantified by ImageJ software. **G**, **H** Autophagy flux assay of HEI-OC1 cells treated with exosomes with endocytosis inhibition by either dynasore (**G**) or cytochalasin D (**H**), and LC3-II (CQ-Ctrl) was quantified by ImageJ software. **I** HEI-OC1 cells were infected with mRFP-GFP-LC3 (tfLC3) and then treated with exosomes with/without dynasore or cytochalasin D. The numbers of autophagosomes (yellow) and autolysosomes (red-only puncta) per cell were quantified. Scale bar, 10 µm. The results were representative of the data generated in at least three independent experiments. The data were presented as mean ± s.d. n.s., not significant; *P < 0.05; **P < 0.01 by Student’s t-test (**B**–**D**) or by one-way ANOVA (**E**–**I**)

Next, we explored whether exosomes increased autophagy activation by promoting endocytosis. First, we investigated whether internalized exosomes could interact with endosomes, endocytosis-associated organelles, in HEI-OC1. Endosomes were labeled with marker EEA1 and co-cultured with exosomess for 1 h. The results showed that most of PKH26-labled exosomes co-localized with endosomes (Fig. 6A). Then, we measured the expression of endocytosis-related genes. The relative expression of endocytic genes in hair cells was significantly upregulated after exosome treatment (Fig. 6B). The expression levels of endocytosis-associated proteins, including EEA1 and CAV2, and the autophagy-related protein LC3 were measured by western blotting, and the results showed that exosome treatment increased the protein levels of EEA1 and CAV2, as well as the LC3-II level, in HEI-OC1 cells (Fig. 6C). Additionally, immunofluorescence was performed to examine the increased EEA1 level by exosomes (Fig. 6D). To demonstrate whether endocytosis is necessary for exosomes to regulate autophagy in hair cells, dynasore (a specific inhibitor of the DNM GTPase) or cytochalasin D (an inhibitor of actin polymerization) was used. First, the efficiency of endocytosis inhibition was assessed by western blotting, which was performed to measure the expression of CAV2 and EEA1 (Additional file 1: Fig. S3A). Next, western blots were performed to evaluate the change of autophagy-associated proteins LC3, SQSTM1/p62 and BECN1 with or without endocytosis inhibition after exosomes treatment. Consistent with the aforementioned results, exosomes increased the LC3-II and BECN1 level while decreased SQSTM1/p62 expression level (Fig. 6E). Whereas, exosomes failed to increase the LC3-II and BECN1 and to decrease SQSTM1/p62 protein levels in cells treated with endocytic inhibitor dynasore or cytochalasin D (Fig. 6E). Then, western blot analysis was performed to assess autophagic flux in exosome-treated hair cells with or without endocytosis inhibited. Consistent with the aforementioned results, exosomes increased the LC3-II level at 4 h, 12 h, and 24 h in hair cells treated with CQ (Fig. 6F), whereas in cells treated with dynasore, the LC3-II protein level decreased over time in hair cells treated with CQ (Fig. 6G). Additionally, cells treated with cytochalasin D displayed a similar phenotype (Fig. 6H). Moreover, a tandem fluorescence mRFP-GFP-LC3 reporter system was used to monitor autophagic flux change after exosomes treatment with or without endocytosis inhibition. The results showed that exosomes significantly mounted the number of red-only puncta, which is consistent with previous data (Fig. 6I). However, exosomes failed to increase the number of red-only puncta or yellow punta after endocytosis being inhibited (Fig. 6I). These data indicated that exosomes activated endocytosis, and inhibition of endocytosis attenuated the autophagic flux that had been increased by exosomes in HEI-OC1 cells. Taken together, these results suggest that exosomes upregulate autophagy by promoting endocytosis in hair cells.

**Fig. 7:**
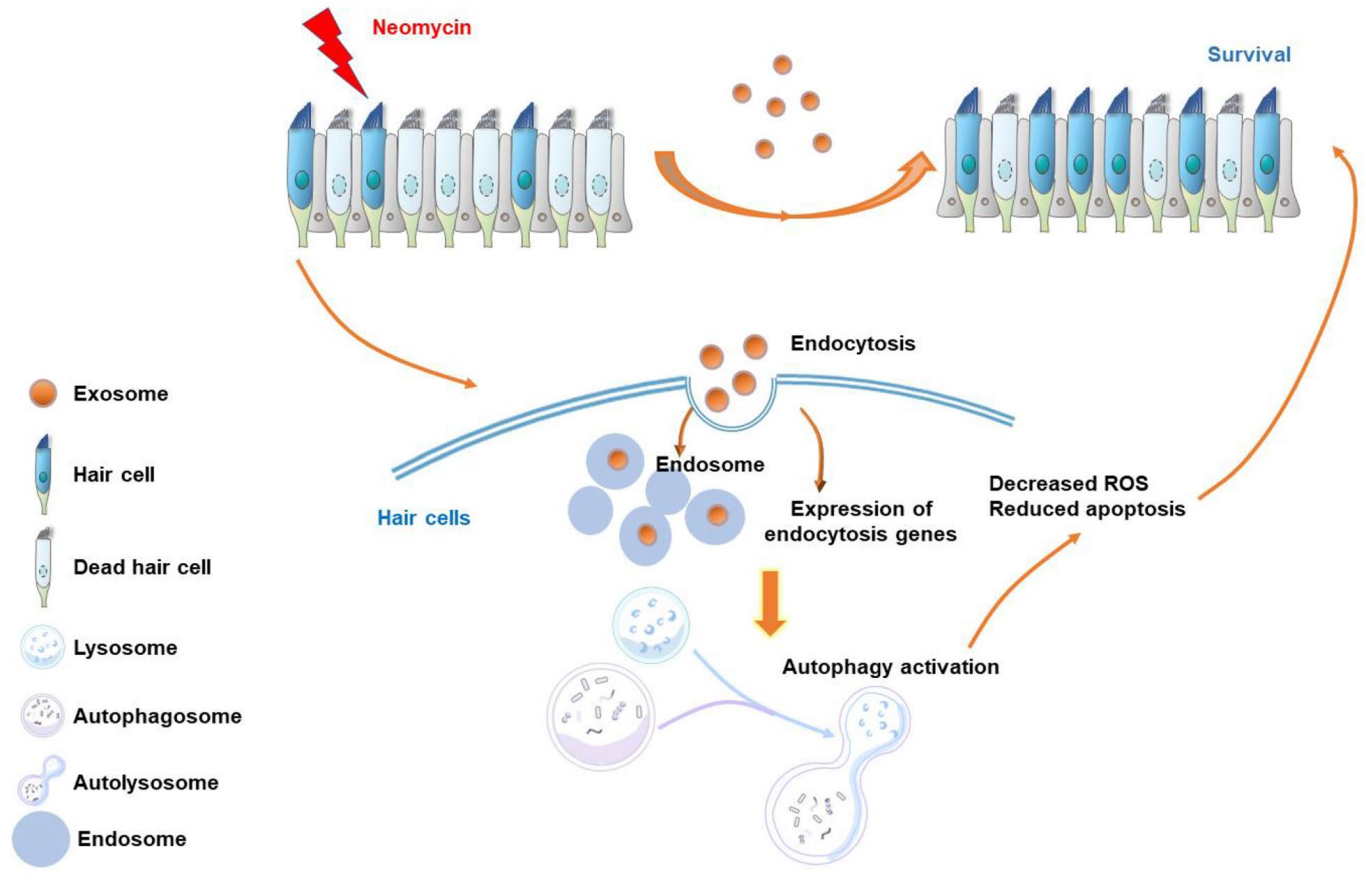
Schema for MSC-derived exosomes protect auditory hair cells from neomycin-induced damage by regulating the autophagy of recipient hair cells. This work describes that exogenously exosomes were internalized by hair cells and subsequently upregulated endocytic gene expression and endosome formation, leading to autophagy activation, which ultimately protect against neomycin-induced damage

In this study, we found that MSC-derived exosomes protected auditory hair cells from neomycin-induced damage via autophagy regulation of recipient hair cells (Fig. 7).

## Discussion

The imbalance between an increasing incidence of SNHL and few treatment options has received considerable attention in the field of disease research, with new biological therapies being developed [3]. Among these therapies, MSC-based therapy is a feasible and safe treatment [7]. Accumulating evidence indicates that MSCs exert their effects through the paracrine signaling mediated by cargo-bearing EVs [25]. EVs have attracted considerable attention because they play key roles in cell-to-cell communication under physiological and pathological conditions [11, 26]. Different-cell-derived EVs exhibit different biological functions because EVs carry different membrane molecules on their surface, in addition to bioactive molecules that are characteristic of the cells from which they are generated [9]. In addition, different types of EVs, including apoptotic bodies, microvesicles, and exosomes, exhibit different biological functions, with the latter being the predominant and most clinically significant type of EV [9]. MSC-derived exosomes exhibit a superior safety profile compared to those derived from other cells because they do not replicate [27] or cause microvascular embolism [28] and can be easily stored without losing their properties [29]. Therefore, extensive research to establish the use of MSC-exosomes for cell-free therapeutic applications in various pathologies [30–32], such as kidney injury [33], cardiovascular disease [34], wound healing [35] and liver failure [36], is ongoing. In the case of inner ear diseases, exosomes derived from supporting cells were initially investigated to ascertain whether their paracrine signaling functions can protect hair cells from neomycin-induced damage [12]. Additionally, an increasing number of reports indicated that MSC-derived exosomes showed the potential to rescue the loss of outer hair cells and repair cochlear damage in a cisplatin-injection model of hearing loss [13, 37]. In this study, we found that exosomes derived from MSCs played a positive otoprotective role by promoting cell survival, reducing mitochondrial ROS levels and reducing the apoptosis rate of hair cells. Our study validates findings showing that MSC-derived exosomes exert protective effects on neomycin-induced damaged cells, making them a potential EV-based alternative therapy for ameliorating SNHL.

Autophagy is an evolutionarily conserved system involving the degradation of cytoplasmic elements and plays a pivotal role in the quality control of proteins and organelles [31, 38, 39]. Autophagy is particularly important in the inner ear, which consists of terminally differentiated cells with very limited regenerative ability in neonates that completely lose their regenerative capacity in adults [15, 16]. This limitation on spontaneous hair cell regeneration contributes to failure to recover hearing ability after hair cells are damaged [3]. In most cases, autophagy is adaptive and inhibits cell dysfunction and death. Interestingly, studies have shown that promoting aminoglycoside delivery to lysosomes through autophagy is a cytoprotective process [40]. Recently, autophagy has emerged as a potential target for treating various forms of SNHL by decreasing the oxidative stress level and apoptosis rate [15, 16]. Our experimental results showed that MSC-derived exosomes markedly increased autophagy activation both in the normal basal state and after neomycin-induced damage in hair cells. Additionally, by measuring autophagic flux, we found that both neomycin and exosome treatments increased the number of autophagosomes and autolysosomes compared to the that in control cells, but these treatment effects exhibited different localization patterns. We found that exosomes significantly promoted more autophagosome-lysosome fusion (increasing more number of red-only puncta) compared to the effect of neomycin-induced autophagy, indicating higher autophagic flux. Moreover, these two treatments may show different mechanism to induce autophagy. Neomycin induced autophagy is accompanied by hair cell damage and increased ROS levels, which indicated cells damage and ROS accumulation may result in promoted autophagy to protect cell from injury. Exosomes may enhance autophagy through an endocytosis-dependent biological process, without affecting the apoptosis and ROC levels of hair cells. Our study provides evidence verifying that targeting autophagy through exosomes is an effective and potential strategy for treating SNHL.

Multiple mechanisms are involved in the modulation of autophagy by exosomes. Exosomes are generally regarded as messengers that carry nucleic acids, proteins, lipids, and metabolites, which participate in intercellular communication and signal transduction in health and disease and affect various aspects of cell biology [9]. However, to deliver their cargo, exosomes need to be internalized by recipient cells either via membrane fusion and/or endocytic uptake [41]. Endocytosis is the initial biological process between exogenous exosomes and recipient cells, and this interaction may mediate some of the biological effects of exosomes in target cells. Interestingly, various studies have shown that endocytosis is critical and necessary for autophagy [42]. Moreover, endosomal components regulate autophagy at multiple stages, constituting an integral component of the autophagic machinery involved in the initiation of degradation, autophagosome maturation and autophagosome-–lysosome fusion [42]. Moreover, studies have indicated that endocytosis and lysosomal delivery sequester aminoglycosides away from cytotoxic targets [40]. In the present study, we found that exosomes activated the endosomal and autophagy pathways in HEI-OC1 cells and that endocytosis was necessary for exosome-mediated autophagy activation. Through our results, we discovered that exosome-driven endocytosis is accompanied by autophagy activation in recipient cells and that endocytosis is necessary for exosomes to upregulate autophagy activation.

A major limitation of this prospective study, in which UC-MSC-exosome therapy protection of auditory hair cells from neomycin-induced damage was investigated, is that only one dose and one route of administration was used. In addition, the use of MSC-exosomes in a novel therapeutic strategy has been limited by challenges, including safety and efficacy concerns hindering clinical research, heterogeneity of exosome composition, and stability during storage and transport. Mechanistically, MSC-exosome treatment mediated anti-apoptotic effects through autophagy activation in an endocytosis-dependent manner, but we cannot exclude the roles of other factors, such as proteins, mRNAs, and noncoding RNAs, due to the complexity of exosome contents.

## Conclusions

Our study sheds light on the efficacy of using exosomes for rescuing neomycin-induced hearing loss and introduces new evidence supporting the possibility that exosomes can be used as therapeutic agents for SNHLs. Considering the association between exosomes and autophagy, our study suggests that autophagy activated by exosomes can be an effective and potential therapeutic strategy for SNHL treatment. Mechanistically, exosome-activated endocytosis is necessary for exosome-mediated autophagy activation. In addition, the underlying mechanism through which exosomes derived from MSCs protect hair cells against damage by regulating autophagy in the inner ear can be applied for auditory protection.

## Methods

### Animals

Animal experiments were approved by the Animal Welfare and Ethics Committee of School of Stomatology, the Fourth Military Medical University following the “Guidelines for the Care and Use of Laboratory Animals”. Wild-type C57BL/6 mice were obtained from the Laboratory Animal Research Center of the Fourth Military Medical University, which were randomly grouped and used for in vivo studies. All mice were housed in a 12-h dark–light cycle at 22 ± 2 °C and 40% humidity and all mice were allowed access to a standard diet. The sample size (n = 6) was decided by “resource equation” method. The confounders were not controlled. No criteria were set to include or exclude animals. The 7-day-old mice were injected subcutaneously with neomycin (dissolve in sterile saline) once per day for five consecutive days at a dose of 200 mg/kg, while the control group was injected with the same amount of sterile saline into the same region without neomycin. The detailed protocol for neomycin administration was given previously [43]. Two days later, exosomes (20 μg in 10 μl PBS) were injected into the mice through the round window niche (RWN) injection, while the control group was injected with the same amount of PBS into the same region without exosomes. Autophagy inhibition in mice was achieved through intraperitoneal (IP) injection of 3-MA at a dose of 30 mg/kg, administrated three times at 24 h before, 2 h before and immediately after RWN treatment. The hearing threshold was evaluated by ABR measurement at P28. After ABR measurement, cochlear tissues were collected for immunofluorescence staining. CAG-RFP-EGFP-LC3 transgenic mice (Jackson Laboratory, 027139) obtained from the Jackson Laboratory were used to test the level of autophagy in the cochlear hair cells. ABR measurement and assessment were performed in a blinded manner. For other experiments, investigators were not blinded since they needed to perform these experiments with different treatments.

### Cell culture, tissue culture and reagents

Umbilical cord mesenchymal stem cells (UC-MSCs) were purchased from Procell (CP-CL11, China) and cultured in α­modified Eagle medium (α-MEM) (Gibco, USA) supplemented with 10% FBS (Sijiqing, China), 2 mM L-glutamine (Invitrogen, USA), 100 U/mL penicillin and 100 U/mL streptomycin (Invitrogen, USA). Cochleae were dissected from P1-3 mice and cultured as previously reported [44]. Briefly, cochleae were dissected and cleaned of surrounding tissue and bone in Hank’s Balanced Salt Solution (Solabio, H1045; China). The cochlear explants were attached to a glass coverslip coated with collagen solution (10X Basal Medium Eagle (BME; Sigma, B9638), 2% sodium carbonate (Sigma, S7795), collagen gel type I (Corning, 354,236), in a 1:1:9 ratio) [45]. The explants were incubated in DMEM/F12 medium supplemented with N2/B27 (Gibco, 17,502,048/17504044, USA) and ampicillin at 37 °C with 5% CO2 overnight prior to each treatment to stabilize them. Neomycin sulfate (Sigma-Aldrich, USA, N6386) was used to damage hair cells at a concentration of 0.5 mM for 24 h. After neomycin was removed, the tissues were treated with exosomes (30 μg/ml for 24 h) or the same amount of PBS. HEI-OC1 cells were cultured at 33 °C with 10% CO2 in high-glucose Dulbecco's Eagle's medium (DMEM) containing 10% FBS without antibiotics. When cells reached a suitable density, neomycin was added to the medium at a final concentration of 2 mM for 24 h to damage the HEI-OC1 cells. After neomycin was removed, the cells were treated with exosomes (30 μg/ml for 24 h) or the same amount of PBS. Chloroquine (HY-17589A), 3-MA (HY-19312), Dynasore (HY-13863), and Cytochalasin D (HY-N6682) were purchased from MCE (USA).

### Western blot

Cells and tissue samples were lysed with RIPA buffer (Beyotime, China) containing protease inhibitor. Protein quantification was performed using a BCA assay (Beyotime, China). Extracted proteins (30 µg) were separated by SDS–polyacrylamide gel electrophoresis (PAGE) and transferred onto PVDF membranes. The membranes were blocked with 5% bovine serum albumin for 1 h at room temperature, followed by incubation with primary antibodies overnight at 4 °C. After washing with Tris-buffered saline-Tween three times, the membranes were incubated with secondary antibodies for 2 h at room temperature. The following primary antibodies were used: Alix (1:1000; Cell Signaling, 92,880, USA), CD9 (1:1000; Abcam, ab236630, USA), CD63 (1:1000; Santa Cruz Biotechnology, sc-5275, USA), CD81 (1:1000; Santa Cruz Biotechnology, sc-9158, USA), GAPDH (1:5000; CWBIO, CW0100, China), Cleaved Caspase-3 (1:1000; Cell Signaling, 9661 s, USA), LC3A/B (1:1000; Cell Signaling 12,741, USA), SQSTMQ/p62 (1:1000; HUABIO HA721171, China), BECN1 (1:1000; Cell Signaling 3495S, USA), CAV(1:1000; Abcam, ab133484, USA), EEA1(1:1000; Abcam, ab109110, USA).

### Isolation and characterization of exosomes

Cell supernatant was first centrifuged at 800 × g for 10 min to remove cells or cell debris. The supernatant was then centrifuged at 16,000 × g for 30 min to remove microvesicles. Next, the supernatant was ultracentrifuged at 150,000 × g for 70 min at 4℃, washed with PBS, and purified by ultracentrifugation at 150,000 × g for additional 70 min. Exosomes derived from MSCs were collected from the bottom of the tube and resuspended in sterile PBS for further use. The protein concertation was measured by BCA kit (Beyotime). The size distribution of exosomes was analyzed using nanoparticle tracking analysis (NTA) with Zeta View PMX 110 (Particle Metrix) and corresponding software, Zeta View 8.04.02. Exosomes were also observed directly under a transmission electron microscopy (TECNAI Spirit, FEI).

### Internalization of exosomes into hair cells in vitro

The cochlear explants were attached to a glass coverslip coated with collagen solution and incubated in DMEM/F12 medium supplemented with N2/B27 and ampicillin at 37 °C with 5% CO2 overnight to stabilize them before each treatment. Exosomes were pre-labeled with the PKH26 Red Fluorescent Cell Linker Kit (Sigma-Aldrich, USA) according to the manufacturer’s instructions and washed in PBS with ultracentrifugation at 150,000 × g for 70 min. PKH26-labeled exosomes at a concentration of 20 μg/ml were then co-cultured with cochlear explants for 8 h or co-cultured with HEI-OC1 for 1 h. After fixation with 4% paraformaldehyde for 20 min at 4 °C, the explants were used for further immunofluorescence staining.

### Immunofluorescence

The samples were fixed in 4% paraformaldehyde (PFA) for 20 min and permeabilized with 0.5% Triton X-100 for 15 min. After washing with PBS, the samples were incubated with primary antibodies at 4 °C overnight, followed by treatment with Cy-3- or FITC-conjugated IgG secondary antibody (1:200; Jackson, USA) for 2 h at room temperature. Cell nuclei were counter-stained with Hoechst 33,342 for 5 min at room temperature. For autophagy flux assay, HEI-OC1 cells were infected with adenoviruses expressing mRFP-GFP-LC3 (MOI 25, Genechem, China) for 72 h and then treated with exosomes and/or Chloroquine (CQ). Images were obtained using the laser scanning confocal microscope (Olympus or Nikon) and quantified. The following primary and secondary antibodies were used in the immunofluorescence studies: Myosin7a (1:400; Proteus biosciences, 25–6790, USA), Cleaved Caspase-3 (1:1000; Cell Signaling, 9661 s, USA), EEA1(1:100; Abcam, ab109110, USA), FITC-conjugated goat anti-rabbit IgG (1:200, Jackson, 111–095-003, USA), and Cy3-conjugated goat anti-rabbit IgG (1:200, Jackson, 111–165-003, USA). Rhodamine-phalloidin (Cytoskeleton, PHDR1, USA), Mito-SOX Red (Invitrogen, M36008, USA), and TUNEL kit (Beyotime, C1090, China) was used to label or measure F-actin, ROS levels and apoptosis according to the manufacturer’s instructions.

### siRNA transfection

HEI-OC1 cells were transfected with siRNA (RiBOBIO) targeting ATG5 using Advanced DNA RNA Transfection Reagent (Zeta Life) according to the manufacturer’s instructions. Next, at 48 h after siRNA transfection, the efficiency of knockdown was determined by western blotting. Subsequent treatments on transfected cells were performed 48 h after transfection.

### Gene expression analysis by real-time qRT-PCR

Total RNA was extracted with TRIzol reagent (Invitrogen, USA), and one microgram of total RNA was reverse transcribed into cDNA with a Prime Script RT reagent kit (TaKaRa, Japan) by the thermal cycler (C1000 Thermal Cycler, Bio-Rad, USA). Real-time RT-PCR was performed with SYBR Green dye and Taq polymerase (TaKaRa, Japan) by the CFX96TM Real-time RT-PCR System (C1000 Touch Thermal Cycler, Bio-Rad, USA). Gene expression was normalized to an internal control GAPDH. The primer sequences are shown in Table 1.

**Table 1:** Primers for real-time PCR

Gene	Description	Primer sequence	Tm (℃)
*Gapdh*	m-Gapdh-F	5ʹ-AGGTCGGTGTGAACGGATTTG-3ʹ	85.5
	m-Gapdh-R	5ʹ-GGGGTCGTTGATGGCAACA-3ʹ	
*Cav2*	m-Cav2-F	5ʹ-CTCACCAGCTCAACTCTCATCTC-3ʹ	88.2
	m-Cav2-R	5ʹ-CAACGTCTGTCACACTCTTCCATA-3ʹ	
*Rab5a*	m- Rab5a -F	5ʹ-CCTTTCTAACCCAAACTGTGTGTCT-3ʹ	83.2
	m- Rab5a -R	5ʹ-CTCGCAAAGGATTCCTCATTTG-3ʹ	
*Rab7a*	m- Rab7a -F	5ʹ-TTGTGTCTCCTCCTCCGTTGAC-3ʹ	86.4
	m- Rab7a -R	5ʹ-CCGCTCCTATTGTGGCTTTGTA-3ʹ	
*Clta*	m- Clta -F	5ʹ-CAAGAAGCGGAGTGGAAAGAAA -3ʹ	86.6
	m- Clta -R	5ʹ-CATAACCAATCAGGTCAGCGAA-3ʹ	
*Ap2a2*	m- Ap2a2-F	5ʹ-GCTGGCATAATACACACCAAAAC-3ʹ	87.7
	m- Ap2a2-R	5ʹ-GAGAGAGACAAGAGAGACGCACA-3ʹ	
*Abc1*	m- Abc1-F	5ʹ-TCGTCTTCCTGTCTGTGTTTGC-3ʹ	90.6
	m- Abc1-R	5ʹ-TGGACCCCGATACCTTGCT-3ʹ	
*Tom1*	m- Tom1-F	5ʹ-CTCAGAGACACCATCCAGACAGAAC-3ʹ	92.3
	m- Tom1-R	5ʹ-GGTGCGGTTCAGTTCCTGTAGTAG-3ʹ	
*Aak1*	m- Aak1-F	5ʹ-CTACTTCACTTTGCCGTTTGGG-3ʹ	84.8
	m- Aak1-R	5ʹ-CCTTTTGTCAGGGTCTGGTTCC-3ʹ	
*Snx2*	m- Snx2-F	5ʹ-CTGTCACTCCCACCACACTCA-3ʹ	83.6
	m- Snx2-R	5ʹ-ATGCCATCACCAACTTTTTCC-3ʹ	

### Flow cytometry

Annexin V and PI assays were conducted to measure the apoptosis of HEI-OC1 cells using the Annexin V-FITC/PI Apoptosis Detection Kit (BD Biosciences, 556,547, USA) according to the manufacturer’s instructions. The cells were collected and resuspended in 100 μL of binding buffer, and stained with 5 μL of FITC labelled Annexin V and 5 μL of PI 15 min at RT (25 °C) in the dark. Add 400 µl of binding buffer to each tube. The stained cells were then analyzed by flow cytometry (Beckman-Coulter, USA).

### Electron microscopy

Cochleae and HEI-OC1 cells were collected and immediately fixed in 2.5% glutaraldehyde for 24 h and then in 1% osmic acid for 2 h, dehydrated with acetone, and embedded in Epon 812. The ultrathin sections were stained with alcoholic uranyl acetate and lead citrate, washed gently with distilled water, and observed with transmission electron microscope (TECNAI Spirit, FEI).

### ABR test

Under light anesthesia with sodium pentobarbital (40 mg/kg), the reference, ground, and active needle electrodes were inserted beneath the skin of the post-measured auricle, the sacrococcygeal region, and the calvaria of each mouse, respectively. ABR was measured using the Tucker-Davis Technology RZ6 system (TuckerDavies Technologies, Gainesville, FL, USA) at 4, 8, 16, 24, and 32 kHz, respectively. ABR waves I and II were monitored to assess thresholds.

### Statistical analysis

Data were expressed as mean ± s.d as indicated. Comparisons between two groups were performed by Student’s t-test, and multiple group comparisons were performed by one-way ANOVA. Tukey’s correction was used when multiple comparisons were performed. *P* values less than 0.05 were considered statistically significant. Graphs and statistical analysis were performed using GraphPad Prism (GraphPad Software 7.0, USA). No animals or data points were excluded.

### Supplementary Information

**Additional file 1: Figure S1.** Exosomes derived from UC-MSCs increased hair cells survival after neomycin-induced damage in a dose-dependent manner. (A) Immunofluorescence staining with myo 7a (green), F-actin (red) and Hoechst (blue) in the apical, middle, and basal turn of cochleae after treated with exosome at different dose following neomycin damage. (B-D) Quantification of myo7a-positive hair cells per 100 μm in the apical(B), middle(C), and basal(D) turn of cochleae of different groups. Scale bar, 20 µm. The results were representative of the data generated in at least three independent experiments and presented as mean ± s.d. n.s., not significant; *P<0.05; **P<0.01 by one-way ANOVA (B-D). **Figure S2.** Inhibition of autophagy by knocking down Atg5 attenuated exosome-mediated otoprotection in HEI-OC1. HEI-OC1 were transfected with 60 nM negative control siRNA (Ctrl) or Atg5 siRNA (si-Atg5) for 48 h before neomycin exposure and/or exosome treatment (A) The expression levels of ATG5, LC3 and SQSTM1/p62 were evaluated by western blot and quantified by ImageJ software. (B) HEI-OC1 cells were labeled with Mito-SOX (red), and the relative fluorescence intensity was quantified after different treatments. Scale bar, 20 µm. (C) TUNEL and Hoechst double staining and (D) Cleaved CASP3 and Hoechst double staining were performed to detect the percentage of apoptotic HEI-OC1 cells after different treatments. Scale bar, 50 µm. (E) Cleaved CASP3 expression level was detected by western blot in HEI-OC1 cells treated with exosomes and/or Atg5 knockdown following neomycin insults and was quantified by ImageJ software. (F) Analysis of apoptotic HEI-OC1 cells by flow cytometry after different treatments. The results were representative of the data generated in at least three independent experiments. The data were presented as mean ± s.d. n.s., not significant; *P<0.05; **P<0.01 by Student’s t-test (A) or one-way ANOVA (B-F). **Figure S3.** The efficiency of endocytosis inhibition by Dynasore and Cytochalasin D. (A) Expression of CAV2 and EEA1 were detected by western blot after pre-treatment with dynasore (80 μM, 4 h) and cytochalasin D (2 μM, 30 min) and were quantified by ImageJ software. The results were representative of the data generated in at least three independent experiments. The data were presented as mean ± s.d. n.s., **P<0.01 by one-way ANOVA (A).

## Data Availability

All data supporting the findings of this study are available within the paper and its Supplementary Information.

## Abbreviations

SNHL: Sensorineural hearing loss
MSC: Mesenchymal stem cell
HSP70: Heat shock protein 70
UC-MSCs: Umbilical cord mesenchymal stem cells
EVs: Extracellular vesicles
TEM: Transmission Electron Microscope
NTA: Nanoparticle tracking analysis
RWN: Round window niche
ABR: Auditory brainstem response
PI: Propidium Iodide
CQ: Chloroquine
3-MA: 3-methyladenine
PI3K: Class III phosphatidylinositol 3-kinase
IP: Intraperitoneal
PFA: Paraformaldehyde
Myo 7a: Myosin 7a
