# Supplementary material for: MSC-derived exosomes protect auditory hair cells from neomycin-induced damage via autophagy regulation
Source: Biol Res. 2024 Jan 13;57:3. doi: 10.1186/s40659-023-00475-w (PMC10787390; doi:10.1186/s40659-023-00475-w)
Supplement: Supplementary file 1 — Additional file 1: Figure S1. Exosomes derived from UC-MSCs increased hair cells survival after neomycin-induced damage in a dose-dependent manner. (A) Immunofluorescence staining with myo 7a (green), F-actin (red) and Hoechst (blue) in the apical, middle, and basal turn of cochleae after treated with exosome at different dose following neomycin damage. (B-D) Quantification of myo7a-positive hair cells per 100 μm in the apical(B), middle(C), and basal(D) turn of cochleae of different groups. Scale bar, 20 µm. The results were representative of the data generated in at least three independent experiments and presented as mean ± s.d. n.s., not significant; *P<0.05; **P<0.01 by one-way ANOVA (B-D). Figure S2. Inhibition of autophagy by knocking down Atg5 attenuated exosome-mediated otoprotection in HEI-OC1. HEI-OC1 were transfected with 60 nM negative control siRNA (Ctrl) or Atg5 siRNA (si-Atg5) for 48 h before neomycin exposure and/or exosome treatment (A) The expression levels of ATG5, LC3 and SQSTM1/p62 were evaluated by western blot and quantified by ImageJ software. (B) HEI-OC1 cells were labeled with Mito-SOX (red), and the relative fluorescence intensity was quantified after different treatments. Scale bar, 20 µm. (C) TUNEL and Hoechst double staining and (D) Cleaved CASP3 and Hoechst double staining were performed to detect the percentage of apoptotic HEI-OC1 cells after different treatments. Scale bar, 50 µm. (E) Cleaved CASP3 expression level was detected by western blot in HEI-OC1 cells treated with exosomes and/or Atg5 knockdown following neomycin insults and was quantified by ImageJ software. (F) Analysis of apoptotic HEI-OC1 cells by flow cytometry after different treatments. The results were representative of the data generated in at least three independent experiments. The data were presented as mean ± s.d. n.s., not significant; *P<0.05; **P<0.01 by Student’s t-test (A) or one-way ANOVA (B-F). Figure S3. The efficiency of endocytosis inhibition by Dyna [file 40659_2023_475_MOESM1_ESM.docx]

**Additional Figures**

**
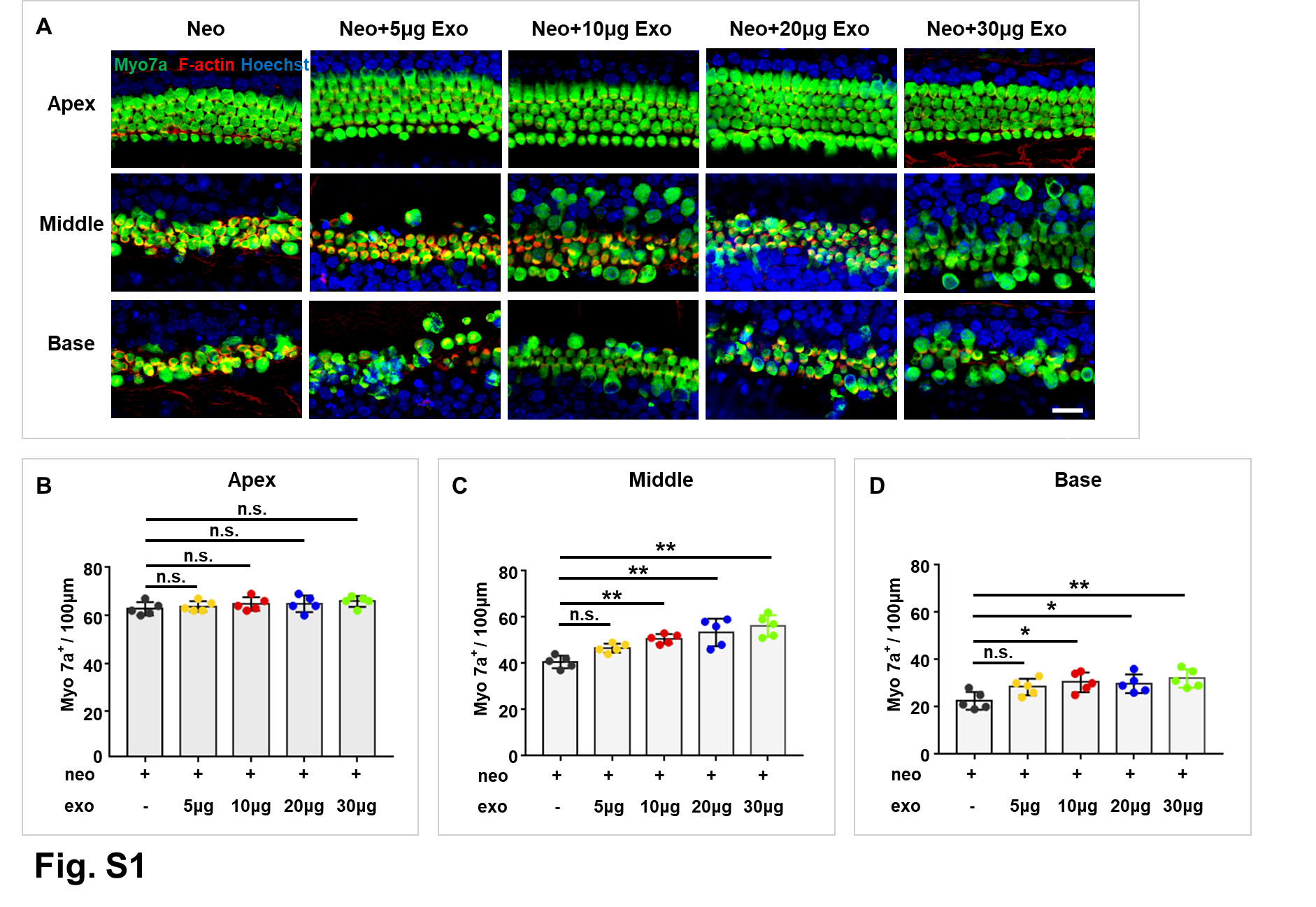
**

**Figure S1. Exosomes derived from UC-MSCs increased hair cells survival after neomycin-induced damage in a dose-dependent manner.** (A) Immunofluorescence staining with myo 7a (green), F-actin (red) and Hoechst (blue) in the apical, middle, and basal turn of cochleae after treated with exosome at different dose following neomycin damage. (B-D) Quantification of myo7a-positive hair cells per 100 μm in the apical(B), middle(C), and basal(D) turn of cochleae of different groups. Scale bar, 20 µm. The results were representative of the data generated in at least three independent experiments and presented as mean ± s.d. n.s., not significant; **P*<0.05; ***P*<0.01 by one-way ANOVA (B-D).


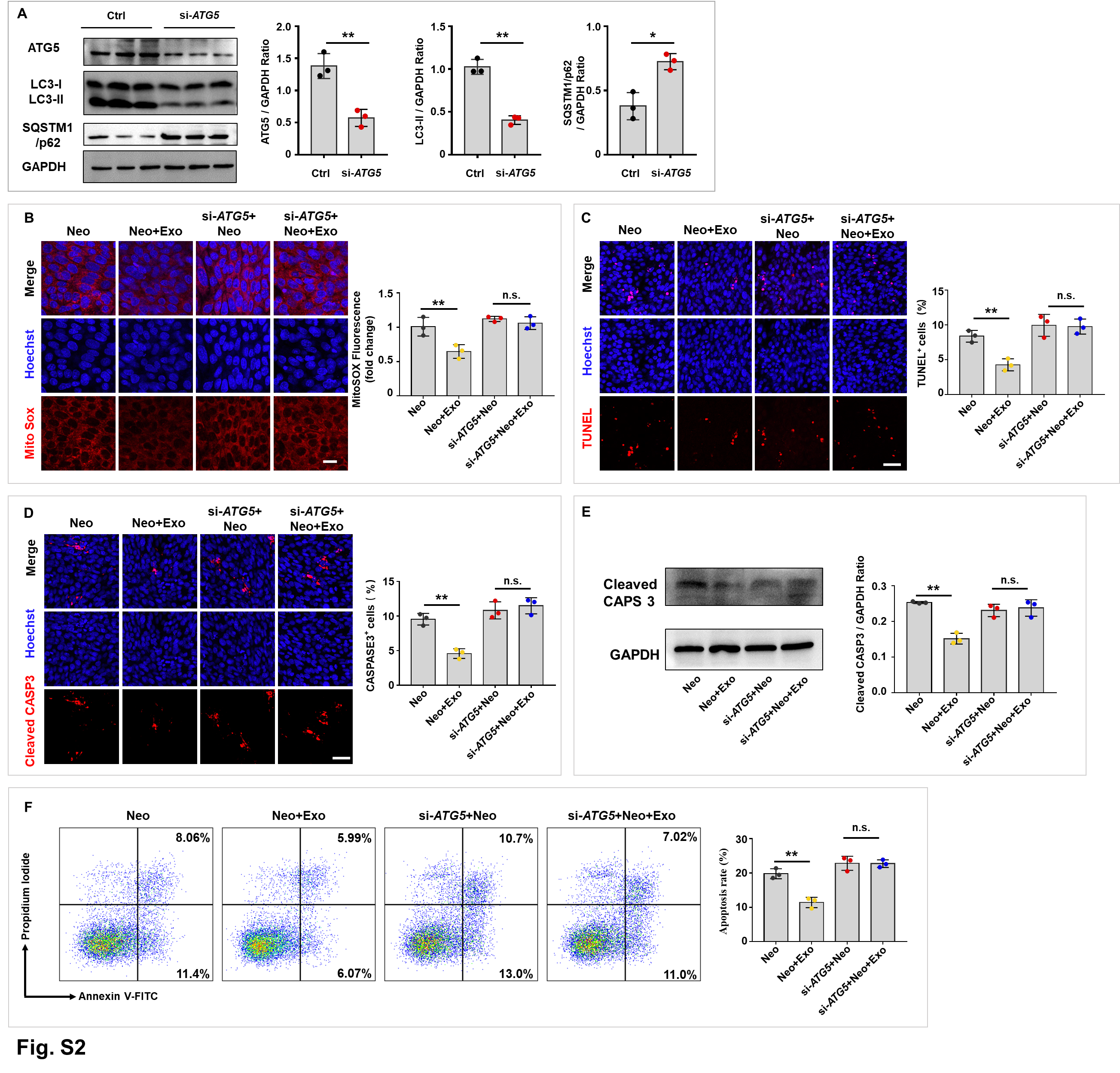


**Figure S2. Inhibition of autophagy by knocking down *Atg5* attenuated exosome-mediated otoprotection in HEI-OC1.** HEI-OC1 were transfected with 60 nM negative control siRNA (Ctrl) or *Atg5* siRNA (si-*Atg5*) for 48 h before neomycin exposure and/or exosome treatment **(A)** The expression levels of ATG5, LC3 and SQSTM1/p62 were evaluated by western blot and quantified by ImageJ software. **(B)** HEI-OC1 cells were labeled with Mito-SOX (red), and the relative fluorescence intensity was quantified after different treatments. Scale bar, 20 µm. **(C)** TUNEL and Hoechst double staining and **(D)** Cleaved CASP3 and Hoechst double staining were performed to detect the percentage of apoptotic HEI-OC1 cells after different treatments. Scale bar, 50 µm. **(E)** Cleaved CASP3 expression level was detected by western blot in HEI-OC1 cells treated with exosomes and/or *Atg5* knockdown following neomycin insults and was quantified by ImageJ software. **(F)** Analysis of apoptotic HEI-OC1 cells by flow cytometry after different treatments. The results were representative of the data generated in at least three independent experiments. The data were presented as mean ± s.d. n.s., not significant; **P*<0.05; ***P*<0.01 by Student’s t-test (A) or one-way ANOVA (B-F).


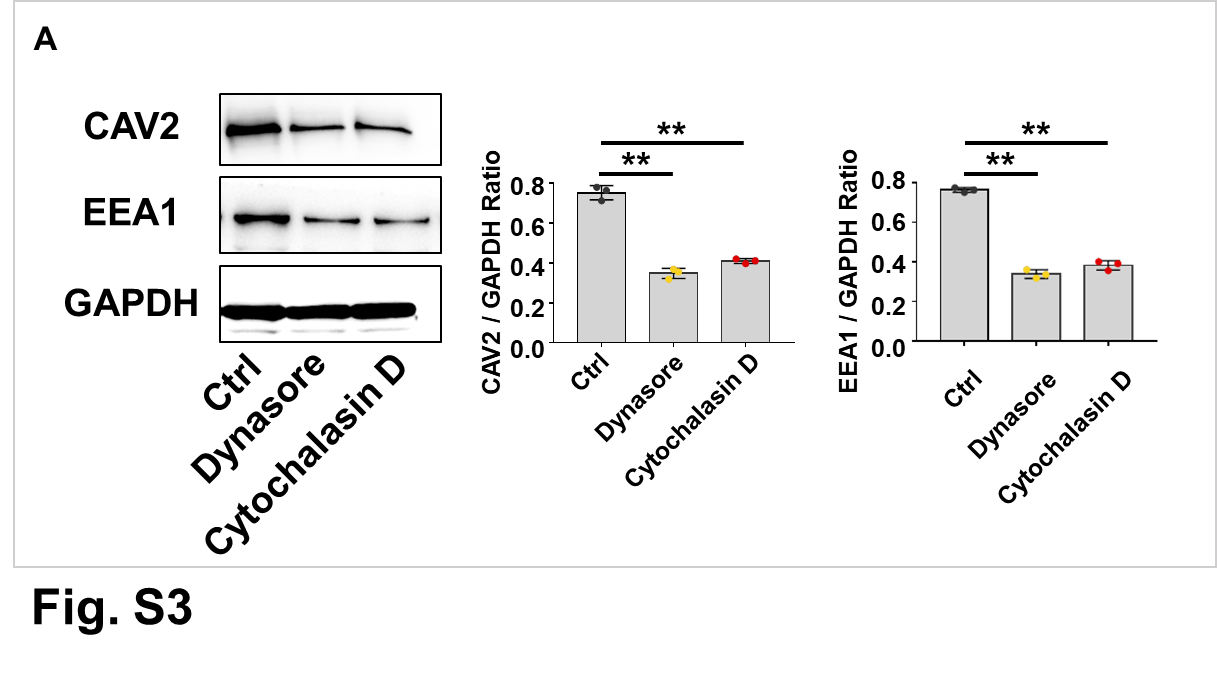


**Figure S2. The efficiency of endocytosis inhibition by Dynasore and Cytochalasin D. (A)** Expression of CAV2 and EEA1 were detected by western blot after pre-treatment with dynasore (80 μM, 4 h) and cytochalasin D (2 μM, 30 min) and were quantified by ImageJ software. The results were representative of the data generated in at least three independent experiments. The data were presented as mean ± s.d. n.s., ***P*<0.01 by one-way ANOVA (A).
